# Mechanisms of ear trauma and reconstructive techniques in 105 consecutive patients

**DOI:** 10.1007/s00405-016-4299-4

**Published:** 2016-10-06

**Authors:** Michail N. Kolodzynski, Moshe Kon, Silvan Egger, Corstiaan C. Breugem

**Affiliations:** Department of Plastic and Reconstructive Surgery, Dutch Center for Ear Reconstruction, University Medical Center Utrecht, Heidelberglaan 100, PO Box 85500, 3508 GA Utrecht, The Netherlands

**Keywords:** Ear reconstruction, Auricular defect, Costal cartilage, Traumatic ear amputation

## Abstract

Acquired auricular deformities may diminish facial esthetics and cause psychological distress. The aim of this article is to provide an overview of the type of injuries and applied reconstructive techniques in a large academic hospital in The Netherlands. A retrospective chart review was conducted for the last 105 patients who underwent auricular reconstruction for an acquired deformity. Data concerning gender, affected side, cause of injury, anatomical region, the previous and further surgeries, type of cartilage, and skin cover used were collected and analyzed. 105 patients were included. Acquired auricular deformities were mainly caused by bite injuries (22 %), traffic accidents (17 %), burns (9.5 %), and post-otoplasty complications (9.5 %). The upper third of the auricle was most often injured (41 %), followed by the entire auricle (19 %). 70 % of cases required reconstruction with costal cartilage. The most common form of cutaneous cover was a postauricular skin flap (40 % of cases). This study gives a complete overview of causes and treatment of acquired auricular deformities. The results are comparable with the results of similar studies found in literature. Bite wounds are the leading cause of acquired auricular injuries. The upper third is most commonly affected. In the largest percentage of reconstructions, costal cartilage and a postauricular flap were used to correct the deformity.

## Introduction

Acquired auricular defects, especially partial defects, are relatively frequent, because of the prominent position of the ear on the side of the head and the delicate skin cover of the complex cartilaginous framework [1, 2]. The auricle with its convex and concave surfaces is designed to collect and amplify sound and direct it to the auditory canal [1]. It is, however, also a ‘decorative’ structure, and although not the most defining part of the face, it can detract from overall esthetics of the face if deformed [3–5]. A deformity or (partial) absence of the auricle can cause psychological problems for the patient [3, 5–7]. The psychological benefit of reconstruction of auricular defects, regardless whether congenital or acquired, has been well documented in the literature [8, 9].

Reconstruction of an acquired auricular deformity is a challenging procedure. It differs in every case and requires tailor-made reconstructions. The aim of any reconstruction is to restore the normal architecture of the auricle. To achieve this, the reconstructed part must have a support structure, usually made out of conchal or costal cartilage, and soft-tissue cover [10–12]. Postauricular skin flaps or advancements are often used, because of easy access and great mobility of the skin. In certain cases, when the skin surrounding the auricle is scarred, tissue expanders or temporoparietal fascia may be used [12, 13].

The aim of this article is to provide an overview of the type of injuries and applied reconstructive techniques using autologous cartilage grafts in a Dutch University Medical Center. Patient characteristics, mechanisms of injury, as well as details about the reconstructions are presented to provide baseline information on the reconstruction of acquired auricular defects.

## Methods

A retrospective chart review was performed for the last 105 patients who underwent a reconstruction of the auricle with a cartilage graft at the Dutch Center for Ear Reconstruction of the University Medical Center Utrecht (UMCU). Patients who were treated for congenital auricular deformities and those reconstructed after tumor excision were excluded. The following characteristics were recorded: name, gender, date of birth, laterality, the etiology of the injury, and the previous surgery of the ear. Each injury was also classified by anatomical region, which was defined as upper third, middle third, and lower third or any combination. This division of the auricle into three zones made it possible to compare our results with those in the literature. Furthermore, the age at reconstruction, the technic of reconstruction, and type of cartilage used and the need for additional cover for the cartilage framework were recorded. Only the largest groups of each analysis will be discussed at length. Smaller groups will be summarized in the text and are visible in the tables. The findings were compared with previously published literature.

The Medical Ethical Committee UMC Utrecht approved this study.

IBM SPSS Statistics 21 (Armonk, NY: IBM Corp.) was used to compile statistics and figures.

## Results

A total of 105 patients, 44 females (42 %) and 61 males (58 %), were included and their records analyzed in this study. The mean age in the female group was 29.7 years (SD 16.12) and 33.2 years (SD 11.65) in the male group. The left ear was affected in 56 cases (54 %), one case did not mention left or right.

### Etiology

The majority of injuries were due to bite injuries (*n* = 24, 23 %). Nine were inflicted by dogs, ten by humans, and five by horses. All victims of horse bites were female, whereas the majority of human bite victims were male (*n* = 9). Dog bites were relatively evenly distributed between both sexes (four women versus five men).

The upper third was most often affected in bite injuries (*n* = 13), followed by middle third injury (*n* = 4). In two cases, the bite resulted in the loss of the entire ear, and in three cases, two-thirds of the ear were lost. The lower third was injured in a single case. Out of these 23 cases of bite injury, 15 were severe enough to require reconstruction with costal cartilage.

In 18 cases (eight women and ten men), the injury to the auricle was caused by a traffic accident, resulting in the total amputation of seven auricles, four upper thirds, and four upper two-thirds. The middle and lower third were affected in one and two cases, respectively. Two of the upper third defects were reconstructed with costal cartilage and one with conchal cartilage. The fourth upper third defect could be reconstructed without the use of a cartilage graft by advancing the helix. Both lower third cases required conchal cartilage only. All twelve remaining injuries (seven total reconstructions, four upper two-thirds, and one middle third) caused by traffic accidents were repaired with costal cartilage.

Burn injuries were the cause in ten cases (three women, seven men). Burns damaged the upper two-thirds in four cases; the upper third in two cases, and the lower third in one case, and led to complete loss of three ears. Two defects, one of the upper third and one of the lower third, could be reconstructed with a conchal graft. The remaining eight cases required costal cartilage grafts.

Ten reconstructions were mandatory following otoplasty; three because of post-operative infection and one because of post-operative hematoma and subsequent infection and cartilage resorption. In the remaining six patients with post-otoplasty deformities, the exact cause could not be found in the records. In at least four cases, the employed otoplasty technique involved scoring of the auricular cartilage; in five cases, the technique used was not mentioned in the records. The upper and middle third were the most common sites for auricular defects following otoplasty, with these areas being affected in four and three cases, respectively. In the other three cases, post-otoplasty complications resulted in one lower third defect, one upper two-thirds defect, and one total auricular loss.

Six auricles (all in female patients) were deformed following a piercing of the upper pole. Reconstruction involved three upper thirds, two upper two-thirds, and one total auricle, all of which were reconstructed with costal cartilage.

In 12 cases, the exact cause of injury could not be identified. In this group of cases, injuries involved the upper third in eight cases, the upper two-thirds in one case, and the lower two-thirds in one case. In two cases, no information was available. Seven out of these twelve cases were reconstructed with costal cartilage; four with conchal cartilage, and in one case, the type of cartilage was not specified.

The remainder of cases had a variety of causes and reconstructions. All information pertaining to these cases can be found in Table 1.

**Table 1:** Causes of injury

Etiology	Total (%)	Female (%)	Male (%)
Bite (total)	23 (22)	9 (8.6)	14 (13)
Dog bite	9 (8.6)	4 (3.8)	5 (4.8)
Horse bite	4 (3.8)	4 (3.8)	–
Human bite	10 (9.5)	1 (0.9)	9 (8.6)
Traffic accident	18 (17)	8 (7.6)	10 (9.5)
Burn	10 (9.5)	3 (2.9)	7 (6.6)
Post-otoplasty complication	10 (9.5)	7 (6.6)	3 (2.9)
Hematoma	1 (0.9)	1 (0.9)	–
Infections	3 (2.9)	–	3 (2.9)
Deformity	6 (5.7)	6 (5.7)	–
Cause unclear	12 (11)	3 (2.9)	9 (8.6)
Post-piercing deformity	6 (5.7)	6 (5.7)	–
Fall	5 (4.8)	2 (1.9)	3 (2.9)
Infection	5 (4.8)	2 (1.9)	3 (2.9)
Occupational accident	4 (3.8)	–	4 (3.8)
Iatrogenic	4 (3.8)	2 (1.9)	2 (1.9)
Glass laceration	3 (2.9)	–	3 (2.9)
Blunt force trauma	2 (1.9)	1 (0.9)	1 (0.9)
Gunshot	1 (0.9)	–	1 (0.9)
Chronic inflammation	1 (0.9)	1 (0.9)	–
Fight	1 (0.9)	–	1 (0.9)
Total	105 (100)	44 (42)	61 (58)

### Previous surgery

Thirty-four patients (32 %) had some form of auricular surgery prior to reconstruction. Thirteen (12 %) underwent the previous reconstruction with the unsatisfactory results. Three patients (2.9 %) had amputated segments of the auricle replanted unsuccessfully. A further three patients (2.9 %) had the denuded cartilage of the lost segment implanted under the skin. None of this cartilage was subsequently used in reconstruction, due to extensive resorption rendering the cartilage useless. In ten cases (9.5 %), the previous surgery, otoplasty, was the cause of a significant deformity, which required reconstruction. A bone-anchored episthesis was placed in two individuals (1.9 %) prior to reconstruction with autologous cartilage. Two cases (1.9 %) had undergone unsuccessful surgeries to improve the shape and definition of the auricle either by deepening the concha or by lipofilling. In a single case, the deformed cartilage was removed in a separate operation prior to reconstruction.

### Anatomical region

**Fig. 1:**
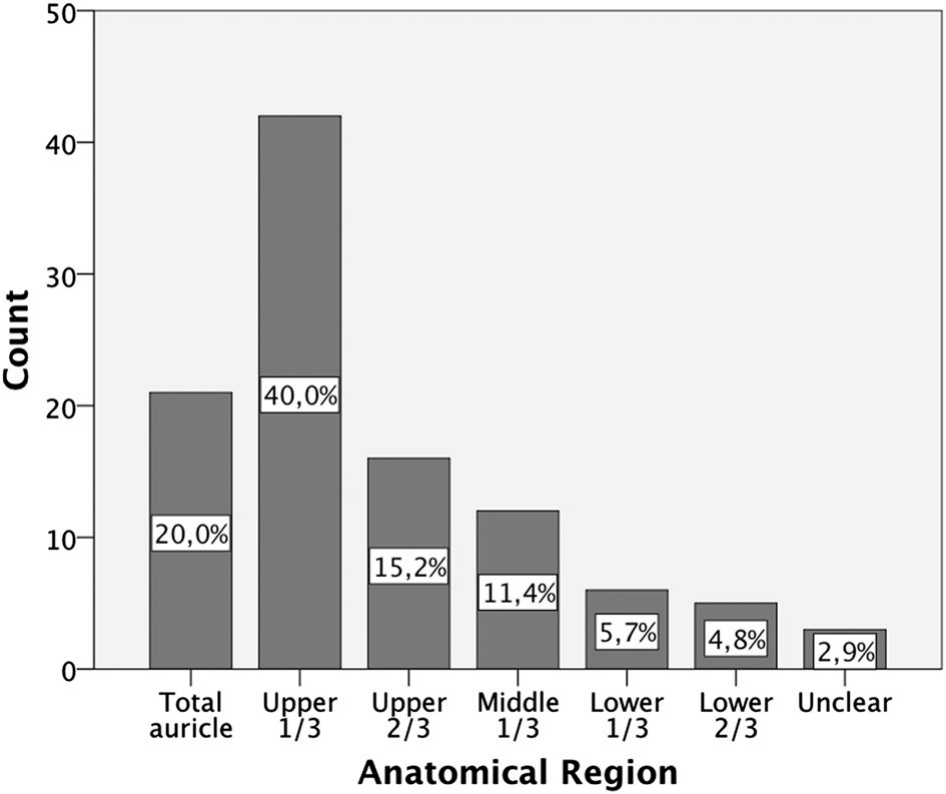
Overview of the affected areas of the ear. *Percentages* are rounded to one decimal

The upper third of the auricle was most often affected by traumatic injury (*n* = 43; 41 %). In another 16 cases, the uppermost two-thirds of the auricle were affected. Twenty ears were lost completely and required total auricular reconstruction. Injuries of the middle third (*n* = 12), the lower third (*n* = 6), and lower two-thirds (*n* = 5) were seen less frequently. Unfortunately, in three of these cases, no information on the anatomical region could be found. An overview of various anatomical regions affected is shown in Fig. 1.

### Type of reconstruction

The majority of ears were reconstructed using costal cartilage (*n* = 73; 70 %). In most cases, the cartilage was harvested from the eighth rib (47 out of 73 cases), often in combination with cartilage from adjacent ribs. Conchal cartilage was used in 28 ears (27 %). This type of cartilage was mainly used in the reconstruction of upper third defects (*n* = 20). Any defect spanning more than a third of the auricle required the use of costal cartilage. Helical advancement techniques, requiring no additional cartilage, were employed in two patients. In another two cases, the type of cartilage could not be found in the records.

Usually, local skin pockets were created to cover the framework (*n* = 53, 51 %). Additional cutaneous cover for the cartilage framework was used in 52 cases (50 %). The (pedicled) postauricular flap was most often used to provide cover (*n* = 21), followed by tissue expansion (*n* = 12) and temporoparietal fascia flap (*n* = 11). The anatomical regions requiring any form of additional cutaneous cover in descending order are upper two-thirds (10/16; 63 %), middle third (7/12; 58 %), upper third (23/43; 53 %), and total auricle (10/20; 50 %). Lower ear injuries required additional cover in 17 % (1 out of 6 cases) and 20 % (1 out of 5 cases), respectively, for lower third and lower two-thirds defects.

The types of injury that required additional cutaneous cover most frequently were burns (7 out of 10; 70 %) and bite injuries (15 out of 23; 65 %). In the group of patients suffering a bite injury to the ear, horse bite victims (*n* = 4) had the highest rate of any form of additional cover used (75 %), followed by human bite victims (7 out of 10; 70 %) and dog bite victims (5 out of 9; 55 %).

74 patients (71 %) underwent mobilization of the ear as a second-stage procedure to achieve normal projection. In 21 cases, a skin graft and additional cartilage were used.

## Discussion

Ear reconstruction remains one of the most challenging procedures encountered by reconstructive surgeons. This is due to the intricate detail and anatomic complexity of the cartilaginous auricular framework and its relationship with its thin soft-tissue envelope. The golden standard is a reconstruction with autologous costal cartilage introduced by Tanzer [14]. This is later expounded by Brent [11] and refined by the work of Nagata and Firmin [15]. This study focused on autologous ear reconstruction after trauma.

Bite injuries were the leading cause of acquired auricular deformities. Subsequent injuries were due to traffic accidents, burns, and complications of otoplasty. Surprisingly, in a country known for its high percentage of bikers, only few cases of auricular trauma were seen due to biking injuries. As shown in Table 2, these results are in line with other publications. Pearl [13] found that 36 out of 50 cases (72 %) over a 4-year period were due to bite injury, which is three times as many than seen in this study. Harris [10] found that bites were the causes of injury in 14 out of 28 cases (50 %). Gault [3] pooled the number of cases due to bite injury with accidents and shootings, so separate analysis of bite injuries was not possible. However, his number of 146 out of 249 cases does suggest that bites were among the leading causes. A literature review of trauma cases from 1980 to 2004 performed by Steffen et al. [16] concerning 74 cases in 56 publications shows that 35 % (*n* = 26) of auricular injuries resulted from bites, mainly caused by dogs (12 %) and humans (23 %).

**Table 2:** Causes of injury compared with other literature

Etiology	Harris [10] (*n* = 28)	Steffen [16] (*n* = 74)	Pearl [13] (*n* = 50)	This study (*n* = 105)
Bite (total)	50 %	35 %	72 %	21.9 %
Dog bite	15 %	12 %	4 %	8.6 %
Horse bite	–	–	2 %	3.8 %
Human bite	35 %	23 %	66 %	9.5 %
Traffic accident	18 %	34 %	12 %	17.1 %
Burn	15 %	–	10 %	9.5 %
Post-otoplasty (total)	11 %	–	–	9.5 %

A study performed by Henry et al. showed that bite injuries are not only the most common cause of acquired auricular defects, but also demonstrated that the ear is the predominant site for facial bite injuries, even more so than the other facial ‘extremity’, the nose [17]. The bite injuries were similarly distributed over the three regions of the ear in Henry et al., as in our study, with the upper third being affected in most cases (49 % compared with 57 % in this article).

The second most leading cause in this study, traffic accident, is also found to be a common cause in the literature. Harris found traffic accidents to be the cause of auricular injury in 18 % (*n* = 5) of cases, compared with 12 % (*n* = 6) found by Pearl and 17 % (*n* = 18) found in this study [10, 13]. Hyckel found a very high incidence of traffic accidents with 13 out of 15 cases (87 %) of trauma to the ear being caused by traffic accidents over a period of 25 years [6]. The incidence for burn injury to the ear found in this article (9.5 %) is lower than those found by Harris (15 %), Gault (12 %), and Pearl (10 %) [3, 10, 13].

Complications of otoplasty are not that common, but can cause a significant damage to the auricle, and are among the leading causes of acquired auricular deformities. In this study, 10 out of 105 (9.5 %) reconstructions were following otoplasty. The numbers and percentages found by Gault, 32 out of 249 cases (13 %), and Harris, 3 out of 27 cases (11 %), confirm the significance of post-otoplasty complications in causing acquired auricular deformities [3, 10]. Literature suggests that deformities are more likely to occur in cutting or scoring the cartilage sculpting techniques and that the result of cartilage sculpting is more difficult to control [18]. Based on these findings, suturing techniques are recommended over sculpting techniques.

Upper third injuries of the auricle were, by far, the most common, followed by total auricle injury, upper two-thirds injury, and middle third injury. In almost 70 %, a costal cartilage graft was mandatory to correct the deformity. Subsequently a mastoid flap also often encountered. Although not the scope of this study, autologous rib cartilage reconstruction can be associated with complications, such as infections, pneumothorax, and chest wall deformities [19].

There are some limitations to this study. The number of patients that could be included was rather limited and it remains unclear whether all patients that underwent reconstruction of an acquired auricular deformity were found. There is a possibility that surgeries and patients were registered under the wrong code and, therefore, could not be found and included for analysis. Data were incomplete in some patients, while in two patients, the records were almost non-existent.

This study focused on ear reconstruction with autologous tissue only. Other options of ear reconstruction are reconstruction with porous polyethylene frameworks [20, 21]; however, this was not used in our unit. Prosthesis camouflage often has successful results in patients who are not candidates for surgical reconstruction or in the elderly patient [22, 23].

More research is needed to investigate patient’s satisfaction after all the different types of surgery. A quality-of-life analysis would be interested among these patients, our future studies will also focus on this topic. We believe that the best treatment for patients with acquired auricular deformities is a reconstruction with costal cartilage, and because of the challenging process, it should be carried out in specialized units performing these operations regularly.

## Conclusion

The ear is prone to trauma due to its prominent location on the side of the head. In the case of acquired auricular deformity or defect, the upper third is most often affected. The leading causes of auricular injury are bite wounds, most often by humans, followed by traffic accidents, burns, and post-otoplasty complications. Most reconstructions of acquired auricular deformity required a costal cartilage graft and a postauricular flap for cover.
